# Which particles to select, and if yes, how many? Subsampling methods for Raman microspectroscopic analysis of very small microplastic

**DOI:** 10.1007/s00216-021-03326-3

**Published:** 2021-05-12

**Authors:** Christian Schwaferts, Patrick Schwaferts, Elisabeth von der Esch, Martin Elsner, Natalia P. Ivleva

**Affiliations:** 1 Institute of Hydrochemistry, Chair of Analytical Chemistry and Water Chemistry, Technical University of Munich, Elisabeth-Winterhalter-Weg 6, 81377 Munich, Germany; 2 Department of Statistics, Methodological Foundations of Statistics and its Applications, Ludwig-Maximilians-Universität München, Ludwigsstraße 33, 80539 Munich, Germany

**Keywords:** Raman microspectroscopy, Microplastic, Nanoplastic, Automation, Chemometrics, Bootstrap

## Abstract

Micro- and nanoplastic contamination is becoming a growing concern for environmental protection and food safety. Therefore, analytical techniques need to produce reliable quantification to ensure proper risk assessment. Raman microspectroscopy (RM) offers identification of single particles, but to ensure that the results are reliable, a certain number of particles has to be analyzed. For larger MP, all particles on the Raman filter can be detected, errors can be quantified, and the minimal sample size can be calculated easily by random sampling. In contrast, very small particles might not all be detected, demanding a window-based analysis of the filter. A bootstrap method is presented to provide an error quantification with confidence intervals from the available window data. In this context, different window selection schemes are evaluated and there is a clear recommendation to employ random (rather than systematically placed) window locations with many small rather than few larger windows. Ultimately, these results are united in a proposed RM measurement algorithm that computes confidence intervals *on-the-fly* during the analysis and, by checking whether given precision requirements are already met, automatically stops if an appropriate number of particles are identified, thus improving efficiency.

**Supplementary Information:**

The online version contains supplementary material available at 10.1007/s00216-021-03326-3.

**Figure:**
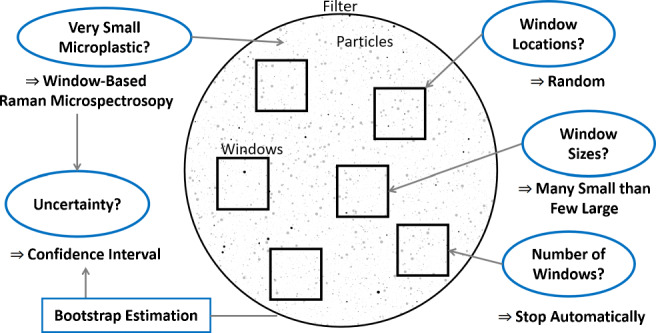
To provide quality control in the MP quantification by Raman microspectroscopy, a window subsampling and bootstrap protocol is presented, which can provide confidence intervals that enable the assessment of the reliability of the data. This is brought together with a proposed on-the-fly algorithm that assesses the precision during the measurement and stops at the optimal point

## Introduction

The ubiquitous plastic contamination in the environment, especially microplastic (MP, 1 μm–1 mm) and, more recently, nanoplastic (< 1 μm), is of great concern and has spawned many efforts to assess the highly diverse aspects of this topic, ranging from its quantity in environmental systems [44] or food (e.g., drinking water [25]) to its toxicity [6]. All of these investigations, however, depend on harmonized analytical methods [16] for which thorough validation is essential. Thus, there are many advances for the several established techniques for MP analysis, which comprise sampling, sample processing, chemical identification, quantification and data processing/reporting [34, 37]. Most of them fall in two main groups: thermoanalytical and spectroscopic methods. The former are based on the analysis of the thermal decomposition products of the polymer by gas-chromatography-mass-spectrometry (GC-MS). Two realizations thereof are pyrolysis-GC-MS [12, 17, 29, 31] and thermoextraction-desorption-GC-MS [8, 9], which give the mass content of different polymers but cannot provide information on the number, size distribution, and morphology of the (plastic) particles. Spectroscopic techniques, on the other hand, comprise mainly Fourier transform infrared spectroscopy [28, 35] and Raman microspectroscopy (RM) [2, 3, 24]. Here, the particles are identified by characteristic vibrational “fingerprint” spectra. This paper focusses on RM, which has been established for the analysis of MP due to the specific data (size distribution, shape, morphology), which are provided by the analysis of individual particles down to 1 μm (and even below) [24].

RM analysis of MP is very time consuming and, until recently, has been also very labor intensive, since the particles had to be measured manually [21]. Hence, substantial advances in its automation have been made, so that there are now several open source [1, 4, 10] and commercial [10, 32, 38] softwares. Currently they are applicable down to the low μm range and are dependent of the maximum image resolution of the RM that was used (von der Esch et al.: 10 μm [10], Brandt et al.: 2–3 μm [4], Ossmann et al.: 1 μm [32]). These automated programs follow the workflow of acquiring an optical image of the filter, particle recognition, RM measurement at resulting coordinates, database matching and result output, where some can control the RM directly [4, 32, 38] and others output the coordinates that have to be passed to the RM control software [1, 10]. There are some applications of Raman imaging, i.e., spectral imaging of entire areas [45]; however, the particle-by-particle approach seems to be preferable [4, 10]. This progress has effected an increase in measured particle numbers in more recent studies (up to several thousand) [2, 39]. Furthermore, the automatic particle recognition removes the operator bias when deciding on which particle to measure and also provides the ratio of MP/non-MP which is an important quantity as opposed to absolute MP number [2].

Parallel to the advances of automated MP quantification, the lower μm range has been targeted. Since the particle number increases exponentially [26], it will become nearly impossible to analyze all particles below a certain size. Thus, a subset of the complete sample has to be selected, which in itself is another sampling. This subsampling — as any sampling — has to satisfy the requirements for correct sampling as laid out by the Theory of Sampling (TOS), demanding that each particle has the same probability to be selected and is not altered [14, 15, 33], and thus, enables a bias-free quality control.

**Fig. 1:**
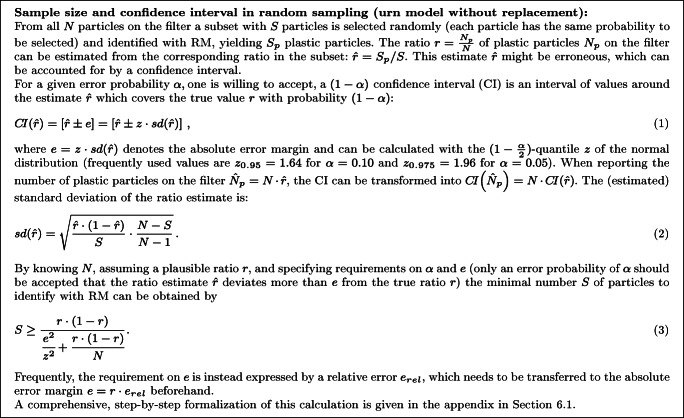
Box containing a summary of sample size and confidence interval calculation in random sampling on a completely imaged filter (urn model without replacement)

The automated routines make it possible to acquire a microscope image of the whole filter and detect all particles (assuming perfect image recognition), thereby the whole sample can be subjected to a random sampling. This equalizes the probability of a particle being selected and makes the spatial structure of the particles on the filter irrelevant. Ergo, this random sampling is a correct sampling and can be modelled statistically (urn model without replacement) to provide an error quantification (via confidence intervals (CI)) and to calculate a minimal sample size such that a certain precision requirement is met [2] (see box in Fig. 1 and the Appendix in Section 6.1.).

For MP, the random sampling is well applicable down to 10 μm [10]. However, for very small MP and especially nanoplastic [11, 36, 40, 46], the complete filter cannot be imaged in a practical manner and the total particle number is not accessible; thus, another subsampling method has to be found. Of course, this problem is not restricted to MP analysis or a specific size range, it is rather universal and relevant whenever particles (points) have to be selected from a two-dimensional surface.

For very small MP (ca. < 10 μm) and even subμ plastic we expect that random sampling may not be feasible due to the following technical concerns.

The measurement time and the computational resources to process the resulting amount of data [7, 27] will increase substantially, due to the fact that smaller particles demand the use of higher magnification objectives (such as 50 ×). For the optical imaging of the complete filter, this leads to a very high number of images that have to be stitched together (our RM would need around 12100 images for a filter with 22 mm diameter, not including image overlap for stitching). It might even be impossible to generate such large images with some commercial RM software, such that some workaround has to be found.

The automated particle measurement critically depends on the RM’s ability to target the particles at their calculated locations. This may become difficult for decreasing particle size, as the deviations by the microscope stage become more and more detrimental. There are multiple parameters that characterize the quality of the microscope stage, which are normed by ISO [23]. From these, there are three that will inhibit the particle identification: accuracy, repeatability and drift. Accuracy describes the discrepancy of the target and the actual position after motion. Repeatability is the accuracy when sequentially positioning. Drift describes the slow temporal component of the position stability. It is usually attributed to temperature fluctuations.

In this application, repeatability [18] is the most pertinent source for deviations, where the positioning system has to be fit to accurately target particles with diameters down to $\sim $ 1 μm. For MP samples with up to 10000 measurement points, this deviation can accumulate to a substantial amount (several μm). Position accuracy and drift over a measurement duration of 2–3 days may, too, be in the same order of magnitude as the particles of concern in this study. Aside the precision of the microscope stage, mosaicking mismatch may also introduce a deviation, since the image stitching is dependent on the availability and recognition of common features in the margins of the single images [7]. The sum of these influences on the deviation is negligible for larger particles (> 10 μm), but prohibits the targeting of very small particles for RM identification, when the images are all taken at once and only then the spectra are acquired.

The acceptable overall positioning error *d*_*err*_ depends on the particle diameter *d* and the laser spot size $d_{laser} = \frac {1.22\cdot \lambda }{N.A.}$, where *λ* is the wavelength of the laser and *N*.*A*. is the numerical aperture of the objective. Its upper bound is $d_{err} < \frac {1}{2}(d + \frac {1.22\cdot \lambda }{N.A.})$, which implies that the laser barely touches the particle. As example, a particle with *d* = 1 µm, measured with a green laser of *λ* = 532 nm and a 50 × objective with *N*.*A*. = 0.7 equates to *d*_*err*_ = 0.96 µm.

With sub-nanometer applications of, e.g., scanning probe microscopy in mind, it is obvious that the quality of high-end positioning systems exceeds the requirement for this problem in some aspects. However, for high-throughput and cost efficient analysis, such high-end technical instrumentation may not be economically feasible.

In this work, we consider the case in which the complete filter cannot be imaged (e.g., in the context of very small MP) and present a window subsampling strategy. Window sampling schemes, although common in MP analysis [5, 13, 19, 22, 32], have the risk of sampling particles incorrectly (cf. TOS) and, furthermore, lack the information on the total particle number, such that corresponding confidence intervals are not analytically accessible. To solve this problem, we describe a bootstrap method to estimate confidence intervals in window sampling schemes and outline that the preferable window scheme uses random window locations. This allows to correctly subsample on the filter and to provide error quantifications via estimated confidence intervals. In the future, this approach can be implemented in the RM measurement process to adjust the sample size with the acquired data *on-the-fly*.

## Window selection schemes

If complete optical imaging of the filter is not feasible (see “Introduction”), RM analysis needs to be restricted to a set of selected windows on the filter. Indeed, this approach is used by many MP laboratories in different varieties (instead of random sampling of all particles). Some studies choose a number of windows (with differing area ratios of the filter) at fixed, arbitrary positions (although referred to as “random”, no randomization was reported and positions seem to be fixed) [13, 22, 32]. There are other window placements that follow a specific arrangement, such as a cross with five [5] and with 19 windows [43]. Other approaches use a spiral [19] or a stratified random window placement [41]. These patterns aim to incorporate potential information about the spatial structure into the window pattern. Thaysen et al. [41] demonstrated the importance of taking the spatial structure of the particles on the filter into account. The resulting information was used to derive a stratified sampling in rings to account for the radial pattern of the particles. However, it is extremely difficult to assess the spatial structure in its entirety, since each statistical analysis only highlights one aspect of the spatial structure.

**Fig. 2:**
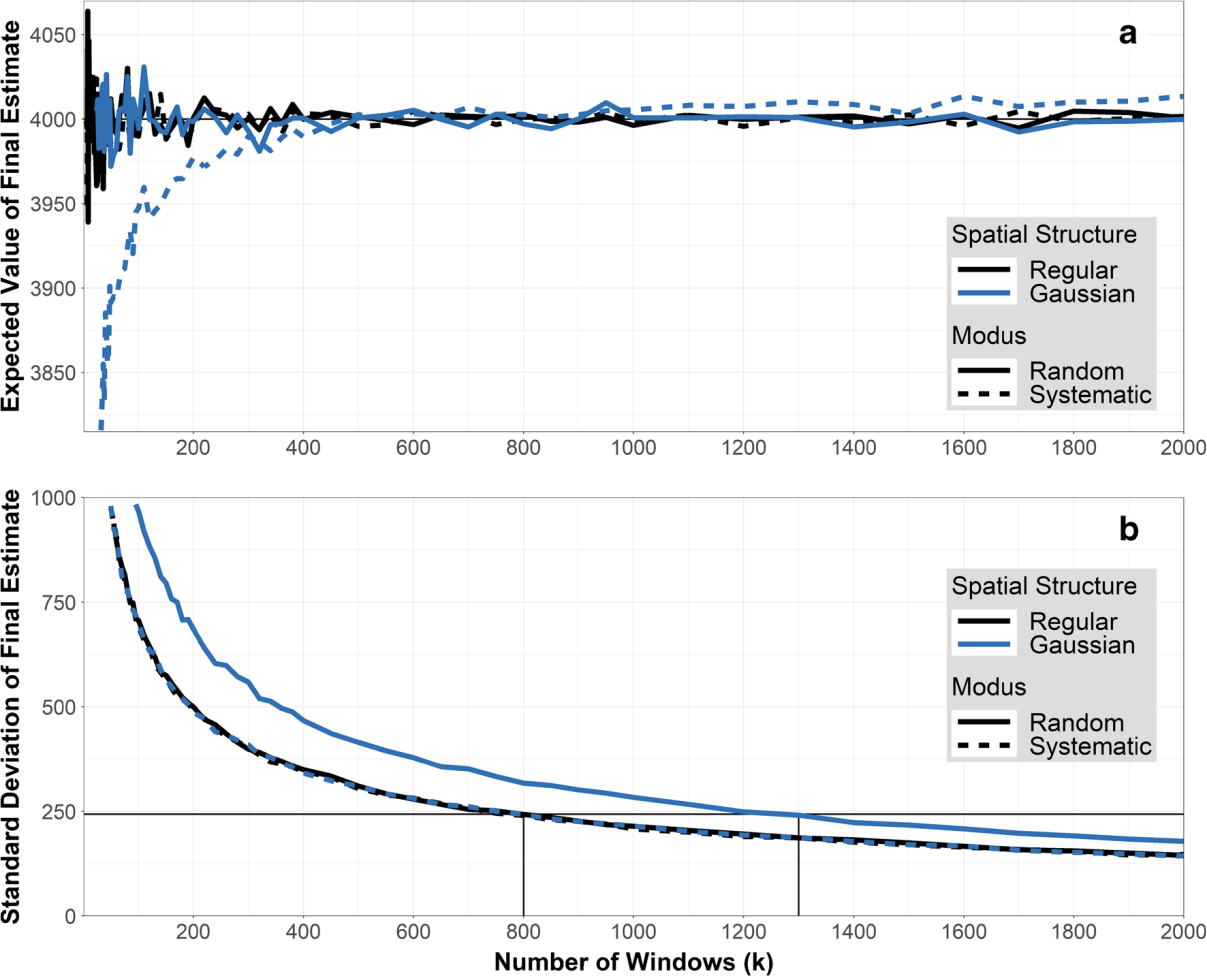
Sampling modus. Artificial filters with regular (black line) and Gaussian (blue line) spatial structure have been analyzed with a random (solid line) and systematic (dotted line) window scheme. Thus, the black and solid line represents random windows on the regular filter. **a** Bias plot: *k* vs. $E(\hat {N}_{p})$. Only systematic windows on the Gaussian filters have a bias (deviation from the true value *N*_*p*_ = 4000 (horizontal black line)). **b** Plot of standard deviation: *k* vs. $sd(\hat {N}_{p})$. Random windows on the Gaussian filter have increased standard deviation. The horizontal black line at $sd(\hat {N}_{p})= 243.2$ corresponds to the precision requirement *e*_*rel*_ = 0.1 and *α* = 0.1. These are met for *k* = 800 and *k* = 1300 windows for regular and Gaussian filters, respectively

Systematic window placement, especially with very few windows, as our example will show, is a critical source of bias (Fig. 2a). Thus, we evaluate these two options: a random window scheme, in which each segment of the filter has equal probability of being investigated, and a systematic scheme with similar distances to the next windows, such that the whole filter is covered by the uniform systematic pattern. We, further, investigate the effect of the window size by comparing windows with a size of 1 and 4 fields of view (FOV), as it might be technically difficult to perform RM on an exceedingly large amount of windows. These will be referred to as *1-FOV sampling* and *4-FOV sampling*, respectively. FOV denotes the size of the microscope image at the respective magnification, which is dependent on the individual microscope (e.g., the RM at the authors lab gives images, i.e., 1-FOV, of 222 μm × 139 μm at 50 × magification). Consequently, *4-FOV* is a 2×2 rectangle of microscope images (444 μm × 278 μm). Adapting the terminology of Minkkinen et al. [30], we denote random vs. systematic windows as *sampling modus* and many small (*1-FOV* ) vs. few large windows (*4-FOV* ) as *sampling type*. Elaborations within this section serve mainly as illustration for the two-dimensional sampling case depicted here and as basis for further elaborations in the subsequent sections. For the general treatment of sampling, see [14, 15, 33].

### Estimation of the number of plastic particles

#### Formalization

Formally, the number *N*_*p*_ of plastic particles of the filter is of interest and can be calculated by considering the number *N* of all particles on the filter and the ratio *r* of plastic particles on the filter: *N*_*p*_ = *N* ⋅ *r*. Both *N* and *r* are unknown and need to be estimated from the window data consisting of *k* windows in total with *W* particles, of which *W*_*p*_ are plastic particles. It is assumed that all particles in the windows will be subjected to RM identification (such that *W* corresponds to the previously used *S*, see Fig. 1), as the number of particles per window is expected to be very low (around 1.6 in the illustrating simulation described below, with 20 000 particles, multiplied by the ratio of window area to filter area). The ratio estimate $\hat {r} = W_{p} / W$ (compare box in Fig. 1) is obtained directly from the window data and the number of particles on the filter can be extrapolated by considering the area *a*(*F*) of the filter in relation to the area *a*(*W*) of the set of windows:

4 $$ \hat{N} = W \cdot \frac{a(F)}{a(W)}  . $$

Together, the number *N*_*p*_ of plastic particles on the filter can be estimated as

5 $$ \hat{N}_{p} = \hat{N} \cdot \hat{r} = W \cdot \frac{a(F)}{a(W)} \cdot \frac{W_{p}}{W} = W_{p} \cdot \frac{a(F)}{a(W)}  . $$

#### Window edge issues

**Fig. 3:**
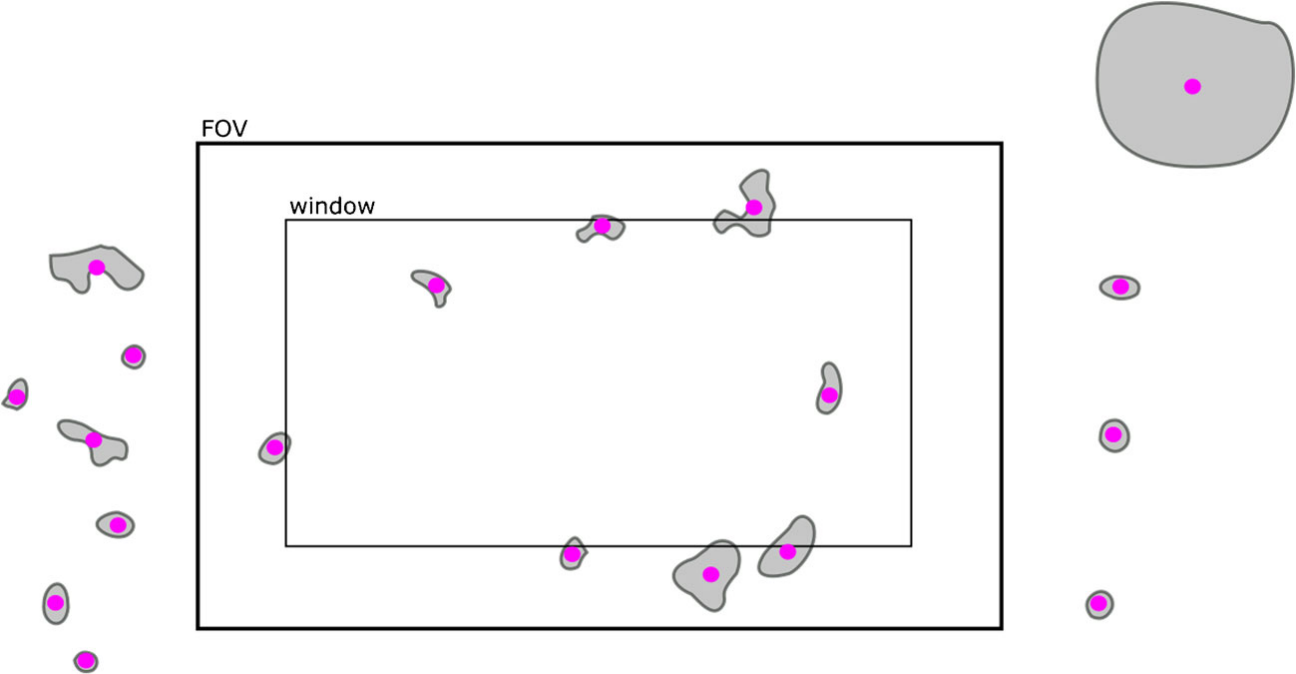
Schematic filter section to illustrate window edge issues: To avoid bias in particle number due to cut off particles, one should only use an inner window of the whole FOV. Then only particles that have their center (magenta) within the inner window, but are contained completely within the outer window (FOV), are counted. So, instead of 8 particles with too low diameter only three particles with correct diameter would be counted

There are some issues if particles overlap with the window edges and are cut off. In this case they will give a false particle size and a distortion in the particles number. Consider Fig. 3, in which many particles overlap the inner window border. Since some are cut off, the 8 recognized particles would truly belong in a slightly larger (outer) window with a larger area, resulting in a lower final estimate for *N*_*p*_ according to Eq. (5). It is even conceivable that a particle is counted twice, if it laps into two closely put windows.

However, a solution to these issues can be achieved by restricting the window-based RM measurement to particles smaller than a certain limit (e.g., 10 μm) and cutting off the radius (i.e., 5 μm) of this limit diameter of each window border (similar to overlap regions in image stitching [7]). Only particles that have their center within this smaller window are of interest. The number of all particles that have their centers within this smaller window, but are completely contained within the larger window (e.g., 3 in the example in Fig. 3) and the area of the smaller window in Eq. (5) will give an unbiased final estimate.

With regard to our target size range, the window size of an image with 50 × magnification is 222 μm × 139 μm, the smaller window would be 212 μm × 129 μm which is still large compared to the size of the particles and should not lead to other serious issues. Should the analysis target larger particles, a larger window would need to be applied (*4-FOV* sampling), since edge issues are smaller with larger windows. In that, edge issues need not be a reason to resign from *1-FOV* sampling when measuring very small microplastic particles.

#### Bias and standard deviation

The final estimate $\hat {N}_{p}$ is a random variable and might be erroneous. Potential errors might be systematic or statistical (random), which can be described by the bias

6 $$ bias\!\left( \hat{N}_{p} \right) = E\!\left( \hat{N}_{p} \right) - N_{p} $$

and the standard deviation $sd \! \left (\hat {N}_{p}\right )$, respectively. The bias of $\hat {N}_{p}$ is the deviation of its expected value $E\!\left (\hat {N}_{p} \right )$ from the true value *N*_*p*_, and should ideally be zero. In that case, the estimate $\hat {N}_{p}$ is unbiased, and if not there is a systematic error that can hardly be controlled in real applications. In fact, the confidence calculation as outlined in Fig. 1 assumes that the estimate $\hat {N}_{p}$ is unbiased, which is the case if random sampling is used.

By assessing both systematic and statistical error for different window sampling schemes, the quality of these schemes can be assessed. However, bias and standard deviation of the final estimate $\hat {N}_{p}$ depend on the spatial influences on the particles on the filter, which are hardly ever fully known, such that explicit formulas cannot be provided. Consequently, obtaining confidence intervals and performing sample size calculations require computational statistical methods.

### Simulation details

To illustrate and elaborate on the questions about sampling modus and type in the next sections, we generated artificial filters, which aimed to resemble a typical preprocessed sample. Since these elaborations target the subsampling on the filter, the other critical aspects of the MP analysis by RM, i.e., particle recognition, or Raman identification, are assumed to be ideal. Method development on these aspects of the analysis is critical to ensure that these assumptions are met and potential error is avoided. A total of *N* = 20000 circular particles, having diameters from 1 μm to 200 μm that follow the power law distribution as reported by Kooi et al. [26], were randomly placed on a circular surface with 22 mm diameter (similar to an Au-coated polycarbonate filter, as used in our laboratory), such that no particles overlap with each other. Out of these particles *r* = 0.2 (20%) were labeled as plastic, resulting in *N*_*p*_ = 4000 plastic particles. This particle number has been chosen with regard to typical filters in the authors’ lab, onto which an appropriate aliquot has to be filtrated such that the filter is not overloaded.

Different influences on the spatial structure of the particles on the filter can be classified as internal and external. Internal influences are interactions between the particles themselves irrespective of their locations on the filter, such as clustering (particles attract each other) or regularity (particles repulse each other), and external influences affect particle (or cluster) locations in general irrespective of potential particle interactions, e.g., particles might tend toward the margin or the center of the filter (for a more comprehensive view on spatial structures see Supplementary Information Section 6.2). Naturally, particles on the filter express regularity as they cannot be in the same place. This is called a *hard core* and signifies an area, in which other particles cannot be located. For the illustration of the external influences, two types of spatial probability distributions were applied in the simulation: a uniform, resulting in a *regular* reference filter without external influence (Supplementary Information Figure S2, left), and a *Gaussian* distribution, resulting in a filter with external influence that collects the particles in the center [20] (Supplementary Information Figure S2, right), similar to the filters by Thaysen et al. [41]. In this manner, 5000 filters have been generated. Window sizes were 222 μm × 139 μm as obtained with the 50 × objective lens (Carl Zeiss AG) of our *alpha 300R* Raman microspectroscope (Witec GmbH, Germany) (and 444 μm × 278 μm for evaluating *4-FOV* sampling, see “Sampling type — smaller vs. larger windows”) and windows were not allowed to overlap with each other.

For each window sampling scheme of interest, the final estimate $\hat {N}_{p}$ was calculated within each of the 5000 simulated filters. All of them (together) allow to estimate its bias $bias\!\left (\hat {N}_{p} \right )$ and standard deviation $sd \! \left (\hat {N}_{p}\right )$ as well as to illustrate effects of external influences on the minimal required sample size *W*, and, thus, the required minimal number *k* of windows. Technical information about the simulation is provided within the Supplementary Information Section ??.

### Sampling modus — random vs. systematic windows

The impact of the sampling modus (random windows vs. systematic windows) on the bias and the standard deviation of the final estimate depends on the number of windows and the actual spatial structure of the particles. Random window locations were allowed such that windows might exceed the margin of the filter, as long as at least some part of the window was still contained within the filter. This ensures that each part of the filter has the same probability to be contained within a window. In contrast, if (random) windows were restricted to be completely within the filter, the outer parts of the filter are underrepresented within the windows, leading to an estimation bias (exemplified and elaborated on in the Supplementary Information in Section 6.4). For systematic windows, a sunflower seed pattern was used, such that the *k* (systematic) windows fill the complete filter with similar distances to their neighbors, trying to cover the area of the filter as uniformly as possible.

Both types of artificial filters (regular and Gaussian) were analyzed using random windows and systematic windows, respectively, for varying numbers of windows *k*. Figure 2a depicts the expected value $E\!\left (\hat {N}_{p} \right )$, which, if unbiased, should equal to *N*_*p*_ = 4000, and Fig. 2b depicts the standard deviation $sd \! \left (\hat {N}_{p}\right )$.

Regarding the bias, Fig. 2a shows that the estimate $\hat {N}_{p}$ is unbiased (the true value is *N*_*p*_ = 4000) in three cases: both of the regular filters and the random windows on the Gaussian filter. The strong oscillation of those lines for small *k* reflects only the simulation variance and will diminish with increasing number of filters analyzed (not just 5000). Only systematic windows on the Gaussian filters lead to a biased estimate. This underestimation is caused by the centralized external influence, which is not adequately represented by the systematic windows. To illustrate this point, imagine a square positioning of 9 windows on the filter. Of these, one lies on the center and 8 lie toward the border. For our centralized, Gaussian particle pattern, this would result in only one window covering a large amount of particles but 8 covering very few, causing an underestimation of the particle number. Similarly, if a spatial structure was present that accumulates particles on the border (as could occur during filtration due to adhesion on the glasswares), this misrepresentation would cause an overestimation of the result.

In general, the strength of this bias depends on the match between the spatial structure of the particles and the pattern of systematic windows used. Within our simulation, this match (Gaussian, centralized structure and sunflower seed arrangement) gets better with increasing number *k* of windows, even nullifying the bias for a certain value of *k* (somewhere between 500 and 1000 windows). This, however, need not be the case in general, and considerable thought should be given to the pattern of window locations, if systematic windows are used. Without any prior information about the spatial structure of the particles, it is difficult to justify the choice of systematic window pattern. In the application of RM analysis of MP, it might however be conceivable to use the spatial information that is gained from the complete filter optical image (for larger MP particles) for the generation of a systematic window scheme for the small size range. Of course, this incorporates the assumption that the small particles behave the same as the large particles. To check this assumption, one might employ an overlap of the size ranges (e.g., complete filter: 10–500 μm and window sampling: 1–50 μm) and compare the results.

It might seem peculiar, that even for Gaussian filters random windows will yield an unbiased estimate. This is because, with random window locations every part of the filter has the same probability to be covered by a window. No matter what the spatial structure looks like and which external influences are present, each characteristic of the spatial structure will be observed with equal probability and no spatial characteristic is systematically missed. This also extends to the case, where plastic particles and non-plastic particles have different characteristics, i.e., if the ratio *r* is not spatially uniform on the filter.

The unbiasedness of random windows, however, comes with an increase in the standard deviation of the estimate. Figure 2b shows that — as expected — the standard deviation decreases with increasing number *k* of windows. This decrease is, again, comparable in three cases: both regular filters and the systematic windows on the Gaussian filter. Only for random windows on the Gaussian filters, the standard deviation is higher, because the procedure of selecting window locations at random introduces additional randomness on the final estimate. The increase in standard deviation for random windows becomes apparent when using the plot to derive a minimal sample size according to predefined precision requirements for the Gaussian filters, as shown in Fig. 2b. Here, precision requirements were specified as *e*_*rel*_ = 0.1 and *α* = 0.1. For *N* = 20000 and *r* = 0.2 this demands the absolute error margin to be smaller than *e* = *N* ⋅ *r* ⋅ *e*_*rel*_ = 400 and, thus, the standard deviation to be smaller than $sd(\hat {N}_{p})= e / z = 243.2$ (with *z* = 1.64, compare box in Fig. 1). In order to obtain this standard deviation (*y*-value) with the Gaussian filters, random windows require *k* = 1300 windows, containing *W* = 2065 particles to identify in total, and systematic windows requires *k* = 800 windows, containing *W* = 1295 particles to identify in total.

Considering systematic windows on the Gaussian filters, it can also be seen that the standard deviation is not affected by the potential bias (see Fig. 2a), emphasizing that both quantities (bias and standard deviation) behave independently and a bias in the data cannot be detected by data processing, as it was laid out in section/chapter “Bias and standard deviation”.

In summary, two characteristics for the sampling modus were observed in this simulation analysis: In the presence of external influences, random windows have an increased standard deviation and systematic windows might yield biased estimates. Although the former increases the probability to obtain a more unrepresentative window sample (due to the increased randomness), this issue can be tackled by increasing the number *k* of windows, however, the latter might introduce a systematic error of unknown size that impairs the representativity of the window sample, which is not controllable in real RM analyses. In that, systematic windows should only be used if their pattern matches well with the spatial structure of the particles. However, as the spatial structures are expected to differ depending on sample origins (marine or limnic waters, drinking waters, processed biota, etc.) and different laboratories with different filtration setups/procedures, such a match needs to be evaluated for each new study. Besides, in contrast to random windows, systematic windows do not allow for an easy way to increase the number of windows adaptively during the analysis, such that a match of the pattern with the spatial structure of the particles can be guaranteed for all numbers of windows.

### Sampling type — smaller vs. larger windows

The impact of the sampling type (*1-FOV* sampling vs. *4-FOV* sampling) on the bias and the standard deviation of the final estimate depends on the number of windows, the actual spatial structure of the particles, and the sampling modus.

**Fig. 4:**
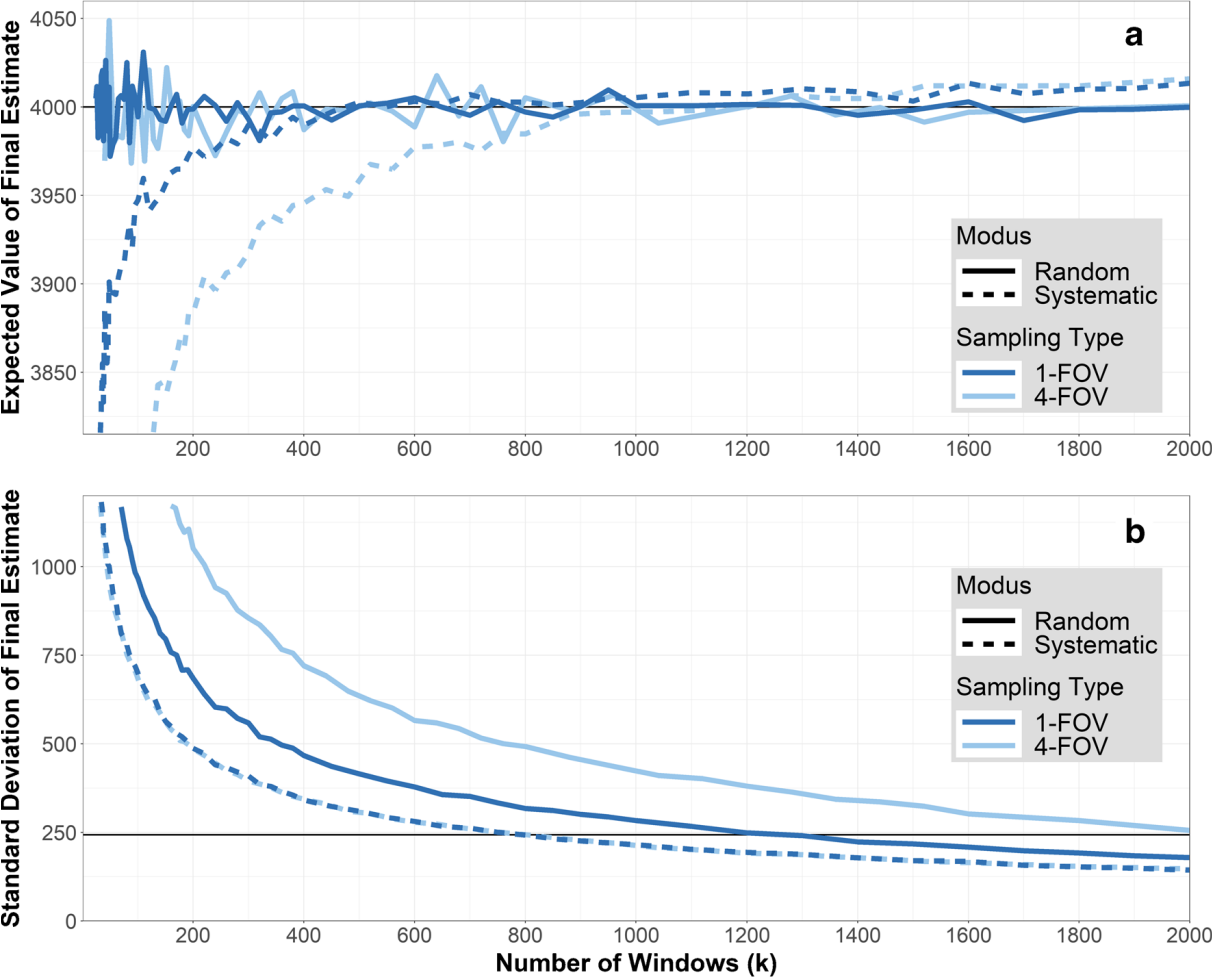
Sampling type. Artificial filters with Gaussian spatial structure have been analyzed with a random and systematic window scheme for both *1-FOV* (dark blue) and *4-FOV* sampling (light blue). The solid line denotes random windows, the dotted line denotes systematic windows. For *4-FOV* sampling, *k* denotes the number of smaller windows needed to obtain the large windows. **a** Bias plot: *k* vs. $E(\hat {N}_{p})$. For systematic windows the bias is more pronounced with *4-FOV* sampling than with *1-FOV* sampling. **b** Plot of standard deviation: *k* vs. $sd(\hat {N}_{p})$. The standard deviation of random windows is higher for *4-FOV* sampling than for *1-FOV* sampling. Analogue to Fig. 2, the black line at $sd(\hat {N}_{p})=243.2$ corresponds to the precision requirements *e*_*rel*_ = 0.1 and *α* = 0.1

As described above (see “Simulation details”), window sizes were 222 μm × 139 μm as obtained with × 50 magnification for *1-FOV* sampling, and 444 μm × 278 μm for *4-FOV* sampling, in order to realistically implement using four smaller windows with × 50 magnification to obtain one larger window. For comparing both sampling types, the number of windows *k* denotes the number of small windows needed to obtain all larger windows in *4-FOV* sampling. As there is no external influence within the regular filters, every window — no matter how its location was determined — has the same distribution of particles or plastic particles. In that, there is no difference between *1-FOV* and *4-FOV* sampling on regular filters, such that results (Fig. 4) focus on the Gaussian filters only. Those were analyzed using both sampling modi (random vs. systematic) and both sampling types (*1-FOV* vs. *4-FOV* sampling) for an increasing number *k* of windows. Again, the figures depict the expected value $E\!\left (\hat {N}_{p} \right )$ (Fig. 4a) and the standard deviation $sd \! \left (\hat {N}_{p}\right )$ (Fig. 4b). Lines for *1-FOV* sampling (dark blue) are the same as in Fig. 2a and 2b.

Figure 4a shows that the bias (deviation from the true value of *N*_*p*_ = 4000) inherent to systematic windows (compare “Sampling modus — random vs. systematic windows”) is larger for *4-FOV* sampling than for *1-FOV* sampling. This is because, *1-FOV* sampling is able to capture the external influence better than *4-FOV* sampling, as more different locations of the filter can be observed. This allows a more comprehensive picture of the different characteristics of external influences, leading to the lower bias in *1-FOV* sampling. Random sampling is still unbiased, independent of the sampling type.

Analogously, Fig. 4b shows that the increase in standard deviation inherent to random windows (compare “Sampling modus — random vs. systematic windows”) is even higher if *4-FOV* sampling is used compared to *1-FOV* sampling. Again, this can be explained because *4-FOV* sampling uses fewer window locations than *1-FOV* sampling, impeding a comprehensive picture of the different characteristics of the external influences.

In summary, *4-FOV* sampling amplifies the problems inherent to random or systematic windows, respectively, in comparison to *1-FOV* sampling, and its effect depends on the strength of the external influences. Therefore, *1-FOV* sampling (i.e., maximizing the number of different window locations) is to be preferred. However, window sizes need also to consider the particle size range of interest (compare “Window edge issues”) as well as processing times, which might be longer for a larger number of smaller windows. Yet, when employing *4-FOV* sampling to reduce processing time, technical limitations for image stitching have to be taken into account (see “Introduction”), and the overall number of particles to identify with RM might be larger than with *1-FOV* sampling, due to the increase in variance, which increases the measurement time.

## Confidence interval via bootstrap

### Theory

If a certain number of windows on the filter were observed, their particles identified, and the final estimate calculated (Eq. (5)), the standard deviation of the final estimate is of interest to calculate a confidence interval. As this depends on the spatial structure of the particles, which is typically not fully known (due to influences of, e.g., the filtration setup, characteristics of the sample or sample treatment), it cannot be obtained analytically (i.e., exactly with a formula). However, bootstrap methods offer a way to estimate this standard deviation and thus the confidence interval.

**Fig. 5:**
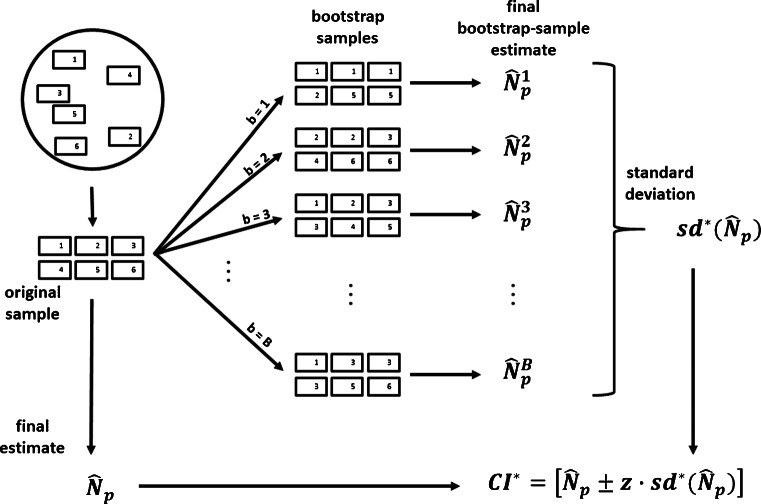
Scheme of bootstrap analysis. From one original window data of the filter (left), *B* samples of the same size are drawn with replacement, each providing a *final bootstrap-sample estimate* for the number of plastic particles $\hat {N}_{p}^{b}$, *b* = 1,...,*B* (middle). These are used to calculate a bootstrap standard deviation *sd*^∗^(*N*_*p*_) (right), which — together with the final estimate $\hat {N}_{p}$ (bottom left) — can be used to estimate a confidence interval *CI*^∗^ (bottom right)

In bootstrap methods (see Fig. 5) new window samples are drawn from the original window sample with replacement (such that in a new sample some original windows might occur more often and other original windows might not occur at all). In a new bootstrap sample, the final estimate might also be calculated (Eq. (5)), thereafter referred to as *final bootstrap-sample estimate*.

Such a bootstrap sample might be drawn many times (e.g., *B* = 5000 times) from the original sample, leading to many *final bootstrap-sample estimates*. The standard deviation of these many *final bootstrap-sample estimates* might then be used as proxy (i.e., a bootstrap-estimate) for the standard deviation of the final estimate, in order to calculate a (bootstrap-based) confidence interval.

Formally, denote the *b* th ($b=1, \dots , B$) *final bootstrap-sample estimate* as $\hat {N}_{p}^{b}$. Their mean and standard deviation are (with ^∗^ indicating the reference to the bootstrap samples)

7 $$ \overline{N_{p}^{*}} = \frac{1}{B} \sum\limits_{b=1}^{B} \hat{N}_{p}^{b} $$

and

8 $$ sd^{*} \!\left( \hat{N}_{p} \right) = \sqrt{ \frac{1}{B} \sum\limits_{b=1}^{B} \left( \hat{N}_{p}^{b} - \overline{N_{p}^{*}} \right)^{2} } , $$

respectively. The latter can be used as proxy for the standard deviation $sd\left (\hat {N}_{p} \right ) = sd^{*}\!\left (\hat {N}_{p} \right )$ of the final estimate, leading to the bootstrap-based confidence interval

9 $$ C\!I^{*} \! \left( \hat{N}_{p} \right) = \left[ \hat{N}_{p} \pm z \cdot sd^{*}\!\left( \hat{N}_{p} \right) \right]  . $$

In every bootstrap method, the representativity of the original (window) sample for the population (filter) is of fundamental importance. Assume, e.g., a Gaussian filter and that all windows are located near the margin of the filter, such that there is hardly any particle in any window. Of course, this window data do not represent the filter well and any bootstrap method might not yield useful results. If the original window sample is less representative for the complete filter, then the bootstrap confidence interval might be longer or shorter than the true confidence interval. While longer confidence intervals provide a conservative error quantification (i.e., the true error probability is smaller than required), smaller confidence intervals provide liberal error quantifications (i.e., the true error probability is larger than required). Typically, the latter is considered far worse than the former, as there is no guarantee that the required limit on the error probability can be kept. As outlined above (“Sampling modus — random vs. systematic windows”), systematic windows might suffer from a bias, impairing the representativity of the window sample in an uncontrollable manner, and random windows express greater variation in the window samples (increasing the likelihood of randomly getting less representative window data), which, however, can be controlled by increasing the number *k* of windows.

### Assessment

**Fig. 6:**
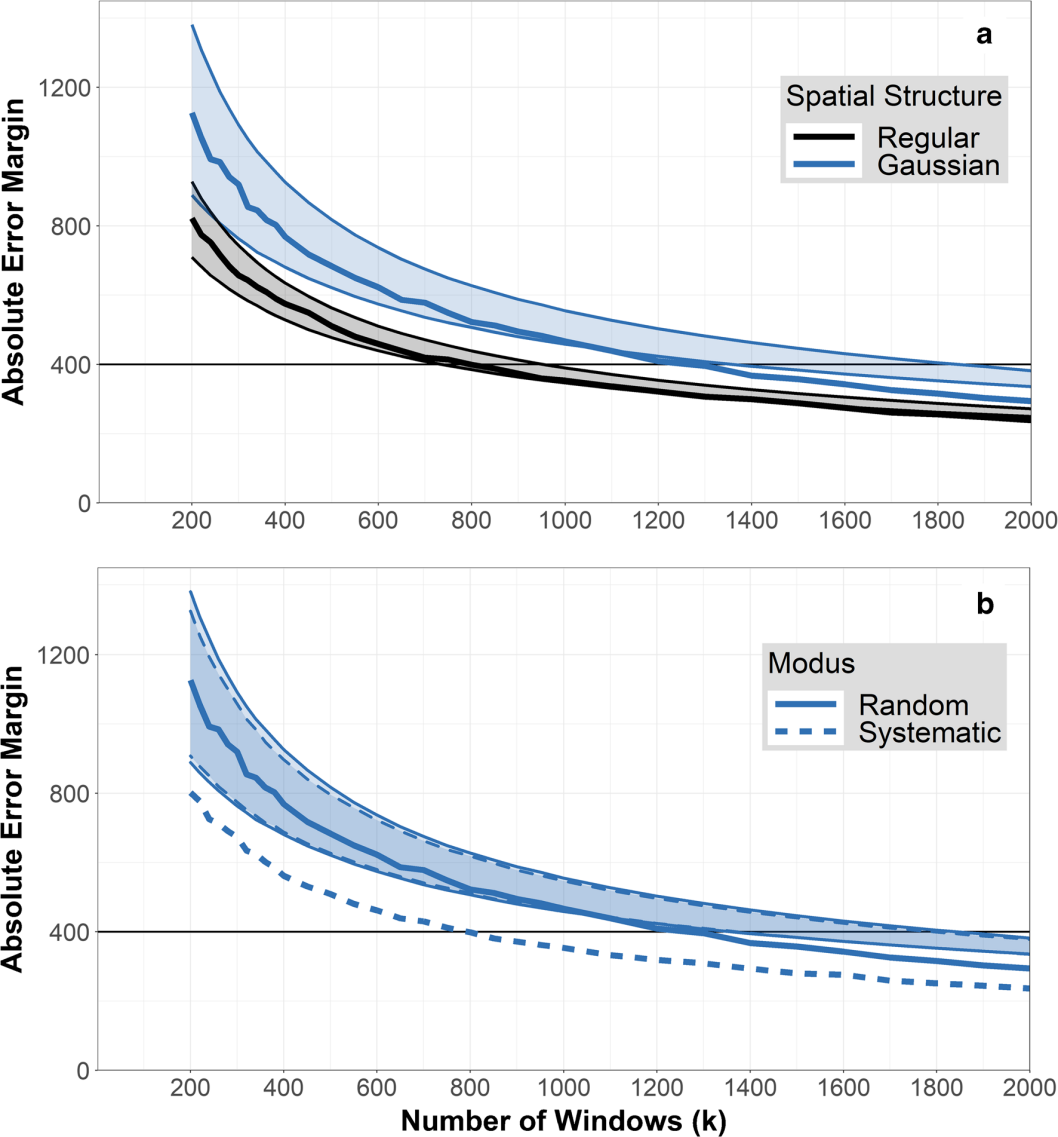
Bootstrap Confidence Intervals. Plots depict lengths of the confidence intervals (absolute error margin *e*) for varying numbers *k* of windows. While the thick lines depict the “true” length of the confidence intervals as obtained by the simulation (see “Sampling modus — random vs. systematic windows” and Fig. 2b), the bands depict estimates of the absolute error margins obtained by the bootstrap methods: For each condition and each number *k* of windows, 5000 bootstrap confidence intervals were estimated and the bands include 90*%* of the corresponding error margin values, excluding the 5*%* lowest and 5*%* largest values. These 90*%*-bands illustrate the additional variation introduced via bootstrap. **a** For both Gaussian (blue) and regular (black) filters, bootstrap confidence intervals tend to be longer, i.e., more conservative, than the “true” confidence interval, especially for larger number *k* of windows (the band deviates upwards from the line). **b** Bootstrap confidence intervals tend to be similar for random (solid lines) and systematic (dashed lines) windows (both bands are similar), although the “true” confidence interval with systematic windows is shorter than with random windows (see “Sampling modus — random vs. systematic windows” and Fig. 2b), illustrating that bootstrap samples are treated as *random* representation of the filter, even if the original windows were placed systematically

A simulation analysis was done to assess the performance of the bootstrap-based confidence interval estimation w.r.t. potential impairments due to a lack of representativity and the additional error introduced by bootstrap estimation. Three conditions were evaluated: random windows for both types of filters (regular and Gaussian) and systematic windows for Gaussian filters (the fourth condition was omitted to save computational resources). Within each condition, for each of the 5000 simulated filters, a varying number *k* of windows were selected. Each of those window data (original sample) were used to estimate a bootstrap-based confidence interval as outlined above, using a given error probability *α* = 0.10. So, for each value of *k* and each condition, 5000 bootstrap-based confidence intervals were obtained (each with *B* = 5000 bootstrap samples), and Fig. 6 shows the 90%-bands of the corresponding absolute error margins (these bands include 90% of these error margin values and exclude the 5% lowest and 5% largest values). Figure 6 also plots the expected absolute error margin obtained with the simulation discussed in section “Sampling modus — random vs. systematic windows” (compare Fig. 2b; values of the absolute error margin relate to those of the standard deviation by $e = z \cdot sd\!\left (\hat {N}_{p} \right )$). This band illustrates the extra variation that is introduced by using a bootstrap method, which — as expected — decreases with increasing number *k* of windows (the band narrows down).

Bootstrap based confidence intervals tend to be longer than the true confidence interval that was obtained with the previous simulation (the band deviates upwards from the line), especially for increasing number *k* of windows. In that, these bootstrap-based confidence intervals tend to be conservative, such that the true error probability might be smaller than the previously specified maximal error probability *α*. Within the Gaussian filters, obtaining a liberal confidence interval becomes unlikely for larger, but reasonable (compare Supplementary Information Section 6.5) numbers of windows *k* > 1300 (the lower limits of the 90*%*-bands surpass the blue line). Although corresponding numbers of particles to identify might be larger with these bootstrap intervals, their conservativeness is a very advantageous property, as it reduces the risk of not being able to meet the requirement on the error probability *α*, which is inherent to a bootstrap method if the original sample is less representative. An analysis of the true error probability of the bootstrap confidence intervals shows that the given error probability *α* could be kept for reasonable numbers *k* of windows (see Fig. S4 in the Supplementary Information).

The 90*%*-bands for random and systematic windows are quite similar. This illustrates an important characteristic of bootstrap methods: New bootstrap window-samples are treated as a *random* window-sample of the filter, even if the original window locations were selected according to a systematic window scheme. Although systematic windows yield a smaller standard deviation than random windows (compare “Sampling modus — random vs. systematic windows”), this is not the case for the bootstrap-estimated standard deviation. In that, when using bootstrap methods to estimate confidence intervals, systematic windows are expected to have no benefit as the standard deviation caused by random windows now compares to systematic windows, and only their downside of generating a potential bias remains.

In summary, bootstrap methods allow to estimate a confidence interval and, thus, to assess the error within the final estimate (which is not possible with formulas), at the cost of introducing an additional source of error. This error, however, seems to shift results in a conservative direction, such that the true error rate might be lower than implied by the results. As a consequence, this might increase the number of particles to identify, but tackle potential representativity issues inherent to bootstrap methods.

Most importantly, a bootstrap confidence interval is only an estimate of the actual (analytically inaccessible) confidence interval and might also be erroneous. It should also be noted that bootstrap methods are not free of critique (for a first overview about bootstrap in general, see [42]). However, in the present case, they might offer a way to check whether the required error margin is roughly reached.

## *On-the-fly* Raman microspectroscopy for very small microplastic

Traditional sample size calculations (if the complete filter is known, see box in Fig. 1) require specifications of the number *N* of particles on the filter and the ratio *r* of plastic particles. The latter is not known prior to the analysis and the former is unknown if window methods are employed. Therefore, those sample size calculations require initial guesses for these values. If those deviate from the true values, then the number of identified particles is too low or too high. Thus, we want to outline an *on-the-fly* algorithm that solves this issue and provides an optimal number of windows. Here, the RM data is analyzed during the measurement by computing a bootstrap confidence interval and assessing whether given precision requirements are already met. If so, the measurement would stop. This avoids having an eventually insufficient sample size or measuring more particles than actually needed.

### Procedure

Aside the precision requirements *α* and *e*, an initial number *k*_*ini*_ of windows should be chosen as starting point for the first bootstrap confidence interval calculation. This is necessary, as mathematical peculiarities might erroneously lead to stop the procedure prematurely, if only few windows were used. In our simulation setup, the average number of particles per window was around 1.6, such that using 100 windows (for *1-FOV* or *4-FOV* ) initially might be a reasonable choice. With regard to chapter/section “Sampling type — smaller vs. larger windows”, the window size should be chosen as small as possible. This number is influenced by *N* and *r*, thus, the sample size calculation of the large particles (random sampling box in Fig. 1) can inform this choice, as this is an optimistic “best case” of the window sampling and displays a lower limit of particles to identify.

**Fig. 7:**
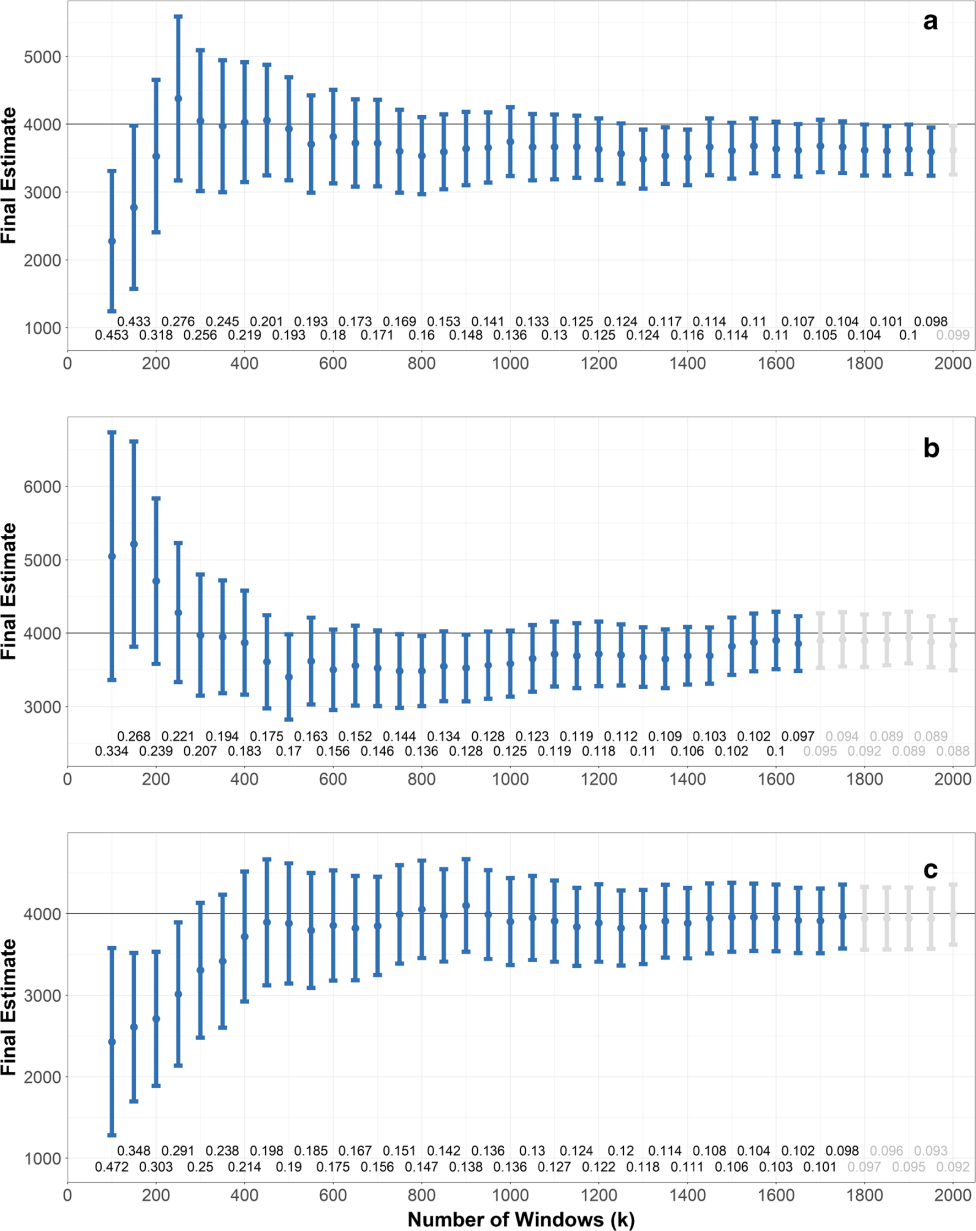
*On-the-fly* procedure: Exemplary runs. For each number *k* of windows, with *k*_*ini*_ = 100 and *k*^+^ = 50, the point represents the final estimate with the vertical bar depicting the bootstrap confidence interval (with *B* = 5000, and *α* = 0.10). The procedure is stopped after the relative error margin (numbers below each confidence interval) fell below *e*_*rel*_ = 0.1, after which the subsequent confidence interval would not be available in a real application but is depicted here for illustrative purposes (grayed out). The black line depicts the true value *N*_*p*_ = 4000. **a** The *on-the-fly* procedure misses the true value of *N*_*p*_ (due to the statistical error). **b** and **c** The algorithm yields a correct result

After identifying the particles in the *k*_*ini*_ windows, if the precision requirements were not met, the number of windows should be increased by an increment *k*^+^ of windows. In theory, it is possible to choose *k*^+^ = 1, but performing too many bootstrap estimations yields the risk of erroneously reaching the stopping criterion (because of the additional randomness inherent to bootstrap methods), especially when approaching the stopping criterion. For the examples in this paper, *k*^+^ = 50 was used. Examples are depicted in Fig. 7.

The total number *B* of bootstrap samples to estimate a confidence interval should be sufficiently large. Within our simulation, we used *B* = 5000, which is comparatively small, to reduce simulation times. In a real application, values as *B* = 10000 or even higher should be easily manageable.

With these specifications, the *on-the-fly* procedure is as follows:

1. As initialization, select *k*_*ini*_ window locations randomly (allowing windows to exceed the filter borders, compare “Sampling modus — random vs. systematic windows”).
2. Detect (consider window edge issues, “Window edge issues”) and analyze all particles within the (newly) chosen windows with RM.
3. Using the complete data set, estimate the number $\hat {N}_{p}$ of microplastic particles on the filter using formula (5).
4. Estimate the standard deviation $sd^{*}\!\left (\hat {N}_{p} \right )$ of this estimate via bootstrap:
  - For *b* = 1,...,*B*:
    Draw a bootstrap sample by drawing *k* windows from the original sample (consisting of the original *k* windows) with replacement.
    Calculate the *final bootstrap-sample estimate* ${N_{p}^{b}}$ within this bootstrap sample using formula (5).
  - Use all *B* *final bootstrap-sample estimates* to calculate their standard deviation $sd^{*}\!\left (\hat {N}_{p} \right )$ (Eq. (8)).
5. For the given significance level *α*, calculate the bootstrap confidence interval (Eq. (9)) and determine the (absolute or relative) error margin.
6. If this (absolute or relative) error margin is larger than the desired (absolute or relative) error margin, select another *k*^+^ windows randomly and return to step 2, else stop the RM analysis.

Within the application of the *on-the-fly* procedure, random windows were used. In theory, it is possible to use systematic windows as well, however, it might be difficult to find a pattern of windows that allows to increase the number of windows sequentially, such that the structure of the systematic windows stays the same. Further, even if such a pattern was found and employed, the benefit in reducing the standard deviation (see “Sampling modus — random vs. systematic windows” and Fig. 2b) is mitigated by using bootstrap methods for estimating confidence intervals (see “Assessment”). Nevertheless, a potential and uncontrollable bias might be introduced by systematic windows, compared to random windows, that might impair the representativity of the window samples in an unknown manner and cannot be controlled by (subsequently) increasing the number of windows. Moreover, it is conceivable that the ratio of MP *r* also exhibits a spatial structure, whose influence is easily circumvented by the random window sampling.

Sample size considerations depend on *N* and *r*, and when using window-based sampling or bootstrap estimation methods this number will be even higher, compared to the random sampling. Our simulation-based illustration uses *N* = 20000 and *r* = 0.20; however, values in real applications cover a very wide range. If the plastic ratios *r* is smaller or the particle number *N* is larger, the sample size may be exceedingly large, such that it may not be possible to identify this amount of particles in reasonable time. In this case, the *on-the-fly* procedure should have a predefined stopping point. Then, of course, it would not meet the precision requirements and it should be considered if some other, less strict precision requirements are agreeable. Furthermore, the experimentator can opt to perform an enrichment step (e.g., density separation) to increase the ratio of MP *r* or, ultimately, decide on a different technique (e.g., thermoanalytical), which of course will trade off the in-depth information of RM for measurement speed (see “Introduction”).

### Examples and assessment

Figure 7 shows three different runs of the *on-the-fly* procedure on the Gaussian filters using *k*_*ini*_ = 100, *k*^+^ = 50, and *B* = 5000. For each iteration, the estimate (point) and its bootstrap-estimated confidence interval (with *α* = 0.10) are depicted in relation to the true value *N*_*p*_ = 4000 (black horizontal line). The last confidence interval that was estimated (in blue) is characterized by a relative error lower than the prespecified requirement of *e*_*rel*_ = 0.10, leading to stop the procedure. Figure 7a depicts a case, in which the final result (estimate plus confidence interval) does not cover the true value, representing an error which might be caused randomly by an unrepresentative window placement. Figure 7b shows an example, where $\hat {N}_{p}$ falls too low but recovers and yields a correct result and Fig. 7c depicts a run that arrives at the true value directly.

In order to assess the performance of the *on-the-fly* procedure, all 5000 filters (of each type) were analyzed (with differing increment sizes to reduce computation time: *k*^+^ = 50 for *k* < 1000 and *k*^+^ = 100 for *k* > 1000). For a significance level of *α* = 0.10, the first *k* with a confidence interval with *e*_*rel*_ < 0.10 was used as result.

The resulting number of windows for regular filters ranged from 650 to 1100 with a mean of 872 ± 108 and for Gaussian filters from 1300 to 2100 with a mean of 1665 ± 69 (respective distributions are depicted in the Supplementary Information Figure S5). This tends to be higher than the minimal number of windows as obtained by the simulation (800 and 1300, respectively, see “Sampling modus — random vs. systematic windows”), which might result from the tendency of the bootstrap estimation to extend confidence intervals in this setup (see “Sampling modus — random vs. systematic windows”).

Of all *on-the-fly* runs on regular and Gaussian filters, only 8.58% and 5.2% yielded confidence intervals that do not cover the true value *N*_*p*_ = 4000, respectively, indicating the conservativeness of bootstrap-based *on-the-fly* results, as an error probability of *α* = 0.1 was allowed in this simulation. In that, the higher window number of bootstrap is counterbalanced by the profit of reducing the true error probability.

In summary, the *on-the-fly* procedure with the bootstrap-based confidence interval provides an algorithmic implementation of error quantification and sample size considerations into window-based RM analyses. By its nature, this adaptive procedure tackles typical specification issues inherent to classic sample size calculations (box in Fig. 1), however, still tending toward conservative results, which, in turn, counteracts potential representativity issues of bootstrap methods. Here, the question about sampling modus is clearly answered: random windows should be employed, as the only benefit of systematic windows (lower standard deviation) seems to get lost by using bootstrap methods.

## Conclusion

Microplastic assessment demands reliable quantification from the analytical techniques, among which RM is able to cover very small particles. Providing quality control to its data has previously been enabled for particles > 10 μm, where random sampling can be applied [10]. Since the smaller particles are more difficult to analyze, a new window-based selection scheme is proposed, for which — as for random sampling — confidence intervals can be obtained. This approach is illustrated on two kinds of spatial structures on the filter: a random distribution with the only stipulation that particles cannot lie within each other, and a Gaussian structure where the particles tend toward the center of the filter. Comparing random and systematic window placement, it is demonstrated that systematic windows are prone to bias, random windows, however, pay for the unbiasedness with increased variance (i.e., standard deviation) and, in turn, number of particles to identify. Further, the importance of using as many small windows as possible rather than few large windows is shown, since the latter amplifies the bias of systematic windows and the variance of random windows, respectively. To achieve a confidence interval for the estimate of the plastic particle number, a bootstrap method was used. Here, the representativity of the sample taken is essential, and random windows are clearly to be preferred. Moreover, due to the random resampling in the bootstrap method, the variance benefit of systematic windows compared to the random windows is lost, nullifying its advantage for this application. Finally, these results were unified in the projection of an *on-the-fly* RM measurement protocol, in which increments of particles are selected, identified and the result instantly subjected to a bootstrap calculation. Its resulting confidence interval then informs the decision to either stop or continue the measurement. This iterative approach solves the problem that an initial sample size calculation requires information on the particle number and MP content, which are only available after the analysis. Therefore, an automated RM analysis could generate MP quantification within a required precision for particles in this very low size range, while also being efficient with measurement time by stopping after the required precision has been reached.

## Electronic supplementary material

Below is the link to the electronic supplementary material.

(PDF 987 KB)
