# Supplementary material for: Which particles to select, and if yes, how many? Subsampling methods for Raman microspectroscopic analysis of very small microplastic
Source: Anal Bioanal Chem. 2021 May 12;413(14):3625–41. doi: 10.1007/s00216-021-03326-3 (PMC8141493; doi:10.1007/s00216-021-03326-3)
Supplement: Supplementary file 1 — (PDF 987 KB) [file 216_2021_3326_MOESM1_ESM.pdf]

6 Supplementary Information

6.1 Random Sampling on the Complete Filter

If every particle is known, then the spatial structure is irrelevant, only the set of particles is relevant. By using random sampling, the error can be controlled and minimized [2]. Thus, random sampling has to be preferred, whenever the possibility. The following section will lay out the formal theory of the confidence interval and sample size calculation for the random sampling. A brief summary has been given in the Box in Figure 1. In the statistical treatment of this problem, a perfect technical implementation of Raman measurement and particle detection shall be assumed – although it still is a current topic, on which diligent work is being performed.

6.1.1 Formalizing the Estimation

Denote the number of particles and of plastic particles on the filter as N and N_p, respectively. Accordingly, the ratio of plastic particles over all particles on the filter is

$$r = \frac{N_p}{N} \,. \tag{10}$$

Only the total number N of particles on the filter is available, but the number N_p of plastic particles on the filter is of interest, which can be calculated by

$$N_p = N \cdot r \,. \tag{11}$$

The ratio r is unknown and usually it is not possible to identify the type of every particle on the filter. Therefore, a subset of all particles on the filter should be selected for RM. Denote the number of particles and plastic particles within this subset as S and S_p, respectively. Both of these quantities are known after RM analysis and the ratio of plastic particles over all particles within this subset can be calculated:

$$r_S = \frac{S_p}{S} \,. \tag{12}$$

This ratio r_S can then be used as an estimate for the ratio r on the filter. In that, we denote the estimate as (an estimate will always be indicated with a hat $\hat{}$ within this section)

$$\hat{r} = r_S \,. \tag{13}$$

By selecting the subset randomly, this estimate $\hat{r}$ is a random variable, and by plugging in this estimate into equation (11), also the quantity of interest becomes a random variable:

$$\hat{N}_p = N \cdot \hat{r} = N \cdot r_S \,. \tag{14}$$

6.1.2 Confidence Interval - Theory

Naturally, this estimate $\hat{N}_p$ might be erroneous and its error can be quantified by considering its standard deviation $sd\left(\hat{N}_p\right)$, which in turn can be derived from the standard deviation $sd(\hat{r})$ of the (random) ratio estimate:

$$sd\left(\hat{N}_p\right) = N \cdot sd(\hat{r}) \,. \tag{15}$$

In that, it suffices to assess the standard deviation of the ratio estimate $\hat{r}$ in order to then quantify the error of the final estimate $\hat{N}_p$.

This standard deviation depends on N, N_p, S, and on the selection scheme of the subset (i.e. which kind of randomness).

The obvious question to ask here is which sampling scheme is the best (i.e. resulting in the lowest standard deviation). Universally agreed on [33,15] and already elaborated on in the field of microplastic [2], this selection should be "completely random", in a sense that each particle on the filter should have the same probability to be selected for RM identification.

By using this random sampling, the selection of particles for RM can be represented by a classical urn model without replacement and the formula for the standard deviation is:

$$sd(\hat{r}) = \sqrt{\frac{r \cdot (1-r)}{S} \cdot \frac{N-S}{N-1}} \,. \tag{16}$$

So, after selecting the subset, doing the RM analysis and calculating the ratio estimate $\hat{r}$ (equation (13)), the standard deviation $sd(\hat{r})$ can be used to determine a confidence interval around the ratio estimate $\hat{r}$. This confidence interval specifies a range of the most plausible values for the ratio estimate $\hat{r}$ and therefore accounts for the estimation uncertainty (in contrast to the point estimate $\hat{r}$ alone).

A confidence interval always refers to a given confidence level $(1-\alpha)$ (where α is the error probability or significance level), such that the probability of the interval covering the true value r is $(1-\alpha)$. Although typical choices are 80%, 90%, 95%, or 99%, the confidence level should be specified according to the actual requirements of the applied context.

The estimate $\hat{r}$ (random variable) is approximately normally distributed (central limit theorem), such that the confidence interval of $\hat{r}$ can be calculated as

$$CI(\hat{r}) = \left[\hat{r} - z_{1-\frac{\alpha}{2}} \cdot sd(\hat{r})\,, \hat{r} + z_{1-\frac{\alpha}{2}} \cdot sd(\hat{r})\right] \,, \tag{17}$$

where $z_{1-\frac{\alpha}{2}}$ is the $(1-\frac{\alpha}{2})$-quantile of the normal distribution (referring to the given confidence level $(1-\alpha)$).

Frequently used values are $z_{0.90} = 1.28$, $z_{0.95} = 1.64$, $z_{0.975} = 1.96$, and $z_{0.995} = 2.58$ for the four α-values mentioned above.

This $(1-\alpha)$ confidence interval of the ratio estimate $\hat{r}$ can then be used to calculate the $(1-\alpha)$ confidence interval of the final estimate (of the number of plastic particles on the filter)

$$CI\left(\hat{N}_p\right) = N \cdot CI(\hat{r}) \ . \tag{18}$$

This confidence interval is then interpreted as being the range of values for $\hat{N}_p$ that covers the true value N_p with probability $(1-\alpha)$, which means that if this procedure of random sampling would be repeated infinitely and confidence intervals would be calculated analogously, then only α of these confidence intervals do not contain the true value N_p.

6.1.3 Confidence Interval - Estimation

Unfortunately, these confidence intervals cannot be calculated as the true ratio r in the formula for $sd(\hat{r})$ (equation (16)) is unknown. Instead, the confidence intervals can only be estimated by using the estimate $\hat{r}$. In that, the estimated standard deviation is

$$\hat{sd}(\hat{r}) = \sqrt{\frac{r_S \cdot (1-r_S)}{S} \cdot \frac{N-S}{N-1}} \tag{19}$$

and the estimated confidence interval of $\hat{r}$ becomes

$$\hat{CI}(\hat{r}) = \left[\hat{r} - z_{1-\frac{\alpha}{2}} \cdot \hat{sd}(\hat{r}) \, , \hat{r} + z_{1-\frac{\alpha}{2}} \cdot \hat{sd}(\hat{r})\right] \ , \tag{20}$$

leading to the estimated confidence interval of the final estimate

$$\hat{CI}\left(\hat{N}_p\right) = N \cdot \hat{CI}(\hat{r}) \ . \tag{21}$$

Instead of reporting solely the point estimate $\hat{N}_p$, this estimated confidence interval $\hat{CI}\left(\hat{N}_p\right)$ should be provided in every microplastic analysis.

6.1.4 Sample Size Calculation

In addition, these considerations about confidence intervals can be used to calculate the required size S of the subset, such that a confidence interval of a given length can be obtained.

As can be seen in equation (17), the confidence interval $\hat{CI}(\hat{r})$ of the ratio estimate $\hat{r}$ is a symmetric interval around the point estimate $\hat{r}$ with "radius"

$$e = z_{1-\frac{\alpha}{2}} \cdot \hat{sd}(\hat{r}) \ , \tag{22}$$

which is frequently denoted by e and referred to as absolute error margin.

In contrast, the relative error margin e_{rel} relates the absolute error margin e to the ratio estimate $\hat{r}$:

$$e_{rel} = \frac{e}{\hat{r}} \ . \tag{23}$$

For example, assume two different confidence intervals around the point estimates $\hat{r} = 0.5$ and $\hat{r} = 0.1$, respectively, both with a relative error of $e_{rel} = 0.1$. In that, the absolute error margins are $e = e_{rel} \cdot \hat{r} = 0.05$ and $e = e_{rel} \cdot \hat{r} = 0.01$, yielding the confidence intervals $[0.45, 0.55]$ and $[0.09, 0.11]$, respectively.

In order to calculate the required number S of particles that should be subjected to RM identification, one needs the following quantities: N, r, $(1-\alpha)$, and e. The first is known, the second is unknown (and no estimate $\hat{r}$ is available prior to the RM identification analysis), and the remaining two should be specified according to the "precision requirements" of the analysis, in a sense that one should state the error probability α, one is willing to accept, that the ratio estimate $\hat{r}$ deviates more than e (absolute error margin) from the true ratio value r. The usual handling of the unknown ratio r is to assume a plausible value and use this assumed ratio.

The required minimum size S can then be calculated as [2]:

$$S \geq \frac{r \cdot (1-r)}{\frac{e^2}{(z_{1-\frac{\alpha}{2}})^2} + \frac{r \cdot (1-r)}{N}} \tag{24}$$

Of course, the assumed ratio r used in this calculation prior to the RM identification process might differ from the estimate $\hat{r}$ (i.e. r_S) that is obtained after RM identification. This might explain that the estimated confidence interval (equation (20)), which is calculated with the ratio estimate $\hat{r}$, might not keep the previously specified "precision requirements" (α and e), which are based on the assumed ratio r.

Nevertheless, it is highly recommended to perform an own sample size calculation prior to RM analysis, as each RM analysis has its own characteristics and requirements. This can be done easily with the following steps:

- Determine the total number N of particles on the filter that might be subjected to RM identification.
- Assume a plausible value for the ratio r of plastic particles among all particles on the filter. If it is too difficult to decide on one single value, try different plausible values. Applying a smaller r increases the sample size but increases the chances that the precision requirements are met.
- State the "precision requirements" (α and e): Only an error probability of α should be accepted that the ratio estimate $\hat{r}$ deviates more than e from the

true ratio r. Frequently, the precision requirement is expressed by a relative error e_{rel}, which needs to be transferred to the absolute margin of error $e = r \cdot e_{rel}$.
- Determine the $z_{1-\frac{\alpha}{2}}$-quantile for the desired maximal error probability α.
- Calculate the minimum sample size S using equation (3).

Of course, all S particles that should be subjected to RM identification have to be selected randomly from all N particles, else the error calculations as outlined above do not hold.

As illustration, table S1 contains results of the sample size calculation for a specific set of precision requirements, i.e. $\alpha = 0.1$ and $e_{rel} = 0.1$. Note that for decreasing r and increasing N the sample size increases, respectively.

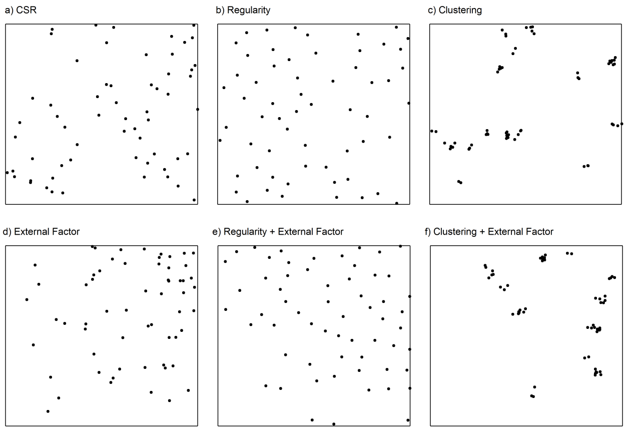

Fig. S1 Influences on Spatial Structures. a) Complete Spatial Randomness: the particle locations have a uniform probability distribution. b) Regularity: particles repulse each other, so that they are less likely to appear close to each other. c) Clustering: particles attract each other, so that they are more likely to appear close to each other. d) External influence: an influence that directs particles to certain locations (here upper right). e) Regular particle distribution with external influence. f) Clustering particles with external influence.

6.2 Spatial Structure of Particle Locations

In many MP laboratories, window sampling is necessary and especially when approaching very small MP it will be mandatory. Here, the influence of the spatial structure of the particles can no longer be evaded by random sampling. This section will discuss the fundamental concepts of spatial structures (especially different types of influences), as laid out in the field of spatial statistics [20].

In principle, there is a so called random point process that can generate spatial structures, which are said to be realizations of this point process. In that, we assume that our observed spatial structure on the filter (e.g. in Figure S2) belongs to a certain, but unknown point process. In the case of MP filtration, the point process would be characterized by the properties of the particles (e.g. propensity for clustering) and the filtration setup (e.g. vacuum pump, fluid dynamic) and the spatial structure would be the actual arrangement of the particles on this one specific filter. Hypothetically, filtering the particle suspension (water sample) again, would give another spatial structure, which is another realization of the same point process.

In spatial statistics different point proceses are discerned [20]. The stereotypical and idealized point process is characterized by *complete spatial randomness* (CSR), such that the location of every point (i.e. particle) is uniformly distributed in the area of interest (i.e. on the filter), which means that every location on the filter has the same probability to be selected as location for a particle (see Figure S1a). With CSR different points might be arbitrarily close to each other.

In general, there are two different types of influences on the spatial structure that deviate it from CSR.

- First, there might be an interaction between the points (particles) themselves (irrespective of their locations on the filter). In that, two different interactions might be distinguished:
  - *Regularity.* In a regular point process, the points repulse each other, such that it is unlikely or impossible for points to be close to other points (Figure S1b). Points or particles are said to have a *hard core*, if it is impossible for other particles to be located within its core (i.e. near surrounding).
  - *Clustering.* When particles attract each other, clusters of particles occur. Cluster locations, however, might still be uniformly distributed on the filter (Figure S1c) or influenced by external influences (Figure S1f).
- Second, point locations might be influenced by external influences (irrespective of the interactions between the points), e.g. points (or cluster locations) might tend towards the margin or the center of the area of interest (see Figure S1d–f).

Having these different types of influences on spatial structures in mind, it appears obvious that spatial structures might be quite complex and their characterization cannot be summarized in one single quantity. In fact, the field of spatial statistics offers a range of different functions, each one only being able to describe a single aspect of a spatial structure [20]. In that, compre-

Table S1 Exemplary values of S for different precision requirements. Columns are N and rows are r.

$\alpha = 0.1$ $e_{rel} = 0.1$	1000	2000	5000	10000	20000	50000	100000	200000	500000	1000000
0.5000	213	239	257	264	267	270	270	271	271	271
0.4000	289	338	376	391	398	403	405	406	406	406
0.3000	387	480	561	594	612	624	628	630	631	631
0.2000	520	703	890	977	1027	1060	1071	1077	1080	1082
0.1000	709	1099	1638	1959	2171	2322	2378	2406	2424	2430
0.0500	838	1440	2535	3396	4090	4662	4890	5012	5089	5115
0.0050	982	1929	4576	8434	14583	25925	34998	42421	48607	51090
0.0005	999	1993	4955	9819	19287	45769	84396	146008	259809	351003

hensively describing or even modeling spatial structures is a very difficult task. "Practically", Pitard even "conclude[s] that the sampling of two-dimensional lots (...) is an unsolvable problem" [33, p. 589].

Interactions between particles influence only the standard deviation of the final estimate, with regularity reducing it and clustering increasing it. Consider the following:

- If particles express regularity (which they do as they have a hard core), it is less likely that in one single window there would be extremely many or extremely few particles compared to when particles would not express regularity. In that, the standard deviation of the number of particles in this window (and therefore also the standard deviation of the final estimate) is lower with regularity compared to without regularity.
- If particles cluster (with random cluster locations), it is more likely that in a single window there would be extremely many (if the window is on a cluster) or extremely few particles (of the window is not on a cluster) compared to when particles do not cluster. Thus, the standard deviation of the number of particles in this window (and therefore the standard deviation of the final estimate) is higher with clustering than without.

A bias would arise if characteristics of the spatial structure would be systematically missed within the observed windows. This is not the case with regularity or clustering per se, as particle locations or cluster locations would still follow a random pattern. Only with an external influence (that affects the locations of particles or cluster, e.g. a vacuum pump vortex) a bias might arise in dependence of the window selection scheme, as illustrated in the paper in Section 2).

Furthermore, concerning the use of the term "homo-/heterogeneity", it seems that those terms are used with strongly differing meanings. In analytical chemistry they can refer to the spatial structure of particles, but also to chemical composition. Spatial statistics uses the term homogeneity in a sense of CSR (Figure S1a) where particle locations have a uniform probability distribution [20]. It, however, appeared to the authors that in analytical chemical texts a "homogeneous" spatial structure typically refers to a regular point process as in Figure S1b. This discrepancy might result from the observation that the distances between the points in the regular spatial structure are relatively similar, thus "homogeneous". Due to this multitude of meanings, we urge for studies to clearly and explicitly denominate the concept of "homo-/heterogeneity" that is employed and we want to emphasize that care has to be taken with those terms in interdisciplinary communication.

6.3 Simulation Details

Exemplary realizations of the artificial filters (regular and gaussian) are depicted in Figure S2. Simulations and analyses were performed in **R** (version 4.0.0) and the code is accessible in an online repository at https://doi.org/10.144 Used packages were boot (version 1.3.24), spatstat (version 1.63.3), ggplot2 (version 3.3.0), ggforce (version 0.3.1), and stringr (version 1.4.0).

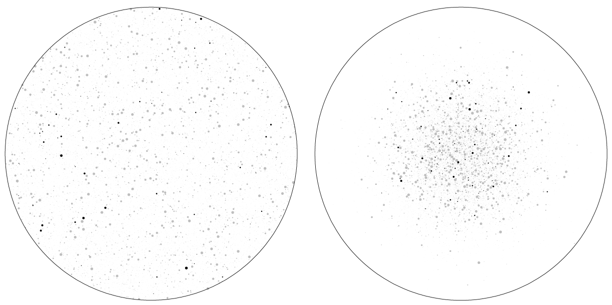

Fig. S2 Example Filters in Simulation. Left: regular filter with particles with hard-core property an uniform probability distribution. Right: gaussian filter with a gaussian-like external influence that directs particles to the center. Each filter contains $N = 20000$ particles of which $r = 0.20$ are plastic particles (black). Particle sizes range from 1 µm to 20 µm and follow a power law as reported by Kooi et al. [26].

6.4 Filter Edge Issues

With the random window scheme it is important that the windows are allowed to overlap the border of the filter. Otherwise, there would be parts of the outer filter that are not properly represented within the windows. This effects an underrepresentation of the border, which can lead to a bias in the final estimate. This is exemplified in Figure S3, where windows did not overlap and, in the presence of an external influence (gaussian filter), the true value $N_p = 4000$ was overestimated by ~ 80 particles.

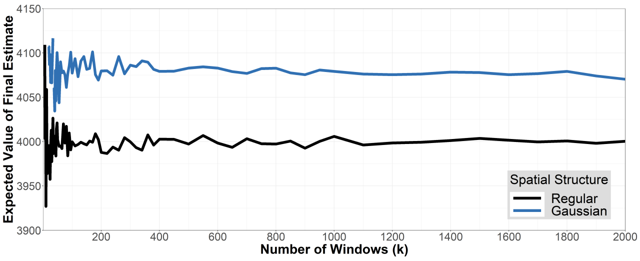

Fig. S3 Filter Edge Issues. Random windows not overlapping the filter border causes an underrepresentation of this part of the filter in the windows. On the two cases of this paper, i.e. regular and gaussian filters, this effects a bias for the gaussian filter (blue) that overestimates the true value $N_p = 4000$.

6.5 Conservativeness in Bootstrap Estimates

By its definition, the $(1-\alpha)$-confidence interval should cover the true value N_p with probability $(1-\alpha)$, i.e. out of all confidence intervals (using the same setup, but different filters with the same external influences), only a ratio of α should be allowed to miss the true value N_p. Figure S4 depicts for different numbers k of windows the ratio of all 5000 bootstrap confidence intervals that do not cover the true value. It shows that for a low number k of windows the bootstrap confidence intervals do not keep the given limit of $\alpha = 0.10$ (black horizontal line), but are conservative for a larger number k of windows.

6.6 Distribution of window number in *on-the-fly*

Within the simulation, actual window numbers after termination of the *on-the-fly* procedure with $e_{rel} = 0.1$ and $\alpha = 0.1$ are depicted in Figure S5 for both regular and gaussian filters.

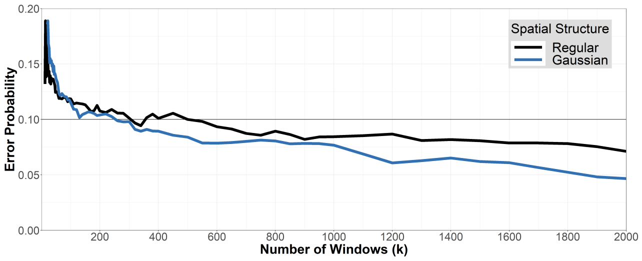

Fig. S4 Conservativeness of Bootstrap Confidence Intervals. The plot depicts the ratio of all 5000 bootstrap confidence interval (with $\alpha = 0.1$) that do not cover the true value of $N_p = 4000$, which estimates the actual error probability α, for both regular and gaussian filters. For typical numbers of windows (in the *on-the-fly* procedure on gaussian filters: $1300 - 2100$, see Figure S5) bootstrap confidence intervals are conservative, as their actual error probability is below the nominated $\alpha = 0.1$.

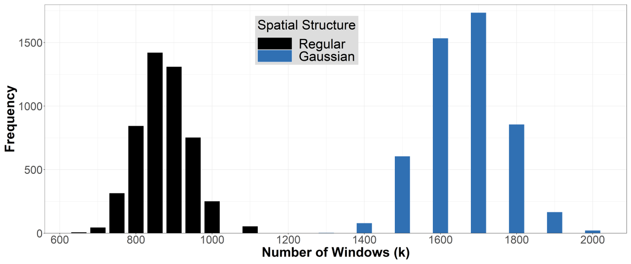

Fig. S5 *On-The-Fly* Procedure: Actual Sample Sizes. Of the 5000 simulated filter, the stopping points k range from 650 to 1100 with $m \pm sd = 1665 \pm 69$ for regular filters and from 1300 to 2100 with $m \pm sd = 872 \pm 108$ for gaussian filters. Window numbers were investigated in increments of 50 for $k \leq 1000$ and 100 for $k \geq 1000$.
